# Incudomalleal joint formation: the roles of apoptosis, migration and downregulation

**DOI:** 10.1186/1471-213X-7-134

**Published:** 2007-12-05

**Authors:** Susan Amin, Eva Matalova, Carol Simpson, Hiroki Yoshida, Abigail S Tucker

**Affiliations:** 1 Department of Craniofacial Development and Orthodontics, King's College London, London, UK; 2 Laboratory of Animal Embryology, Institute of Animal Physiology and Genetics, v.v.i. Academy of Sciences, Brno, Czech Republic; 3 Department of Physiology and Pathophysiology, University of Veterinary and Pharmaceutical Sciences, Brno, Czech Republic; 4 Division of Molecular and Cellular Immunoscience, Department of Biomolecular Sciences, Faculty of Medicine, Saga University, Saga, Japan; 5 Department of Reconstructive Sciences, Centre for Regenerative Medicine and Skeletal Development, University of Connecticut Health Centre, Farmington, CT, USA

## Abstract

**Background:**

The middle ear of mammals is composed of three endochondrial ossicles, the stapes, incus and malleus. Joints link the malleus to the incus and the incus to the stapes. In the mouse the first arch derived malleus and incus are formed from a single *Sox9 *and *Type II collagen *expressing condensation that later subdivides to give rise to two separate ossicles. In contrast the stapes forms from a separate condensation derived from the second branchial arch. Fusion of the malleus and incus is observed in a number of human syndromes and results in conductive hearing loss. Understanding how this joint forms during normal development is thus an important step in furthering our understanding of such defects.

**Results:**

We show that the developing incudomalleal joint is characterised by a lack of proliferation and discrete areas of apoptosis. Apoptosis has been suggested to aid in the removal of pre-cartilaginous cells from the joint region, allowing for the physical separation of the cartilaginous elements, however, we show that joint initiation is unaffected by blocking apoptosis. There is also no evidence of cell migration out of the presumptive joint region, as observed by labelling of joint and ossicle cells in culture. Using *Type II collagen *lacZ reporter mice, however, it is evident that cells in the presumptive joint region remain in place and downregulate cartilage markers.

**Conclusion:**

The malleus and incus first appear as a single united condensation expressing early cartilage markers. The incudomalleal joint region forms by cells in the presumptive joint region switching off cartilage markers and turning on joint markers. Failure in this process may result in fusion of this joint, as observed in human syndromes such as Branchio-Oto-Renal Syndrome or Treacher Collins Syndrome.

## Background

In the mouse the malleus and incus of the middle ear initially develop as a single element that expresses cartilage markers such as *Type II collagen *and *Sox9*. This united structure then subdivides to form the two ossicles divided by the incudomalleal joint [1,2]. This early joint region is free of *Type II collagen *or *Sox9 *expressing cells and expresses joint markers such as *Gdf5 *[1]. *Sox9 *has been shown to upregulate *Type II collagen *expression, and overexpression of *Sox9 *leads to ectopic cartilage formation [3,4]. Loss of *Sox9 *and *Type II collagen*, therefore, is thought to play an important role in formation of the joint.

The formation of three ossicles in the middle ear (malleus, incus and stapes) is a characteristic of mammals. During the course of evolution the primary jaw articulation of non-mammalian vertebrates was replaced by a second articulation between two membranous bones, the squamosal and dentary [5]. Studies involving comparative anatomy, embryology and paleontology have suggested that the primary jaw articulation, along with the hyomandibular (columella in chick and reptiles) were incorporated into the middle ear to form a three ossicle chain. Using this criteria, the malleus is homologous to the articular part of Meckel's cartilage, the incus is homologous to the quadrate/palatoquadrate, and the stapes is homologous to the hypomandibular [5,6]. The incudomalleal joint is therefore homologous to the primary jaw joint. Homology has been confirmed by investigating expression of genes such as *Bapx1 (Nkx3.2)*, which is specifically expressed in the primary jaw joint of *Xenopus*, zebrafish and chick and in the incudomalleal joint in mammals [7-10]. Like the incudomalleal joint, the primary jaw joint divides two initially continuous elements expressing Type II collagen, the quadrate and articular part of Meckel's cartilage [9]. These two cartilages then separate to form the articulation point for the upper and lower jaws.

In a variety of human syndromes, such as Treacher Collins and Branchio-Oto-Renal (BOR) syndrome, the malleus and incus are often fused resulting in conductive hearing loss [11-14]. The development of this joint is therefore essential to ensure correct hearing. It is thus of interest to examine what happens to these presumptive joint cells that are initially fated to become cartilage.

In a variety of limb joints, apoptosis has been observed within the interzone at the centre of the developing joint [15]. Such programmed cell death was postulated to account for the loss of the cartilage lineage cells within the forming joint, leading to the separation of skeletal elements [16-20]. Given this data from the limb, we wished to investigate the role of apoptosis in early joint formation in the middle ear.

Apoptosis can be mediated by distinct pathways initiating from within (intrinsic) or outside (extrinsic) the cell. Both pathways involve signalling via a family of cysteine proteases named caspases [21]. Caspase machinery becomes activated in a cascade manner starting with apical caspases, such as Caspase 8 and Caspase 9, leading to cleavage of effector caspases, such as Caspase 3. *Caspase 8 *mutants die at E11.5 due to cardiovascular abnormalities [22], but *Caspase 9 *and *Caspase 3 *mutant mice survive up to and past birth depending on the strain [23-26]. These mutants display a strikingly similar phenotype, characterised by a prominent brain malformation caused by defective apoptosis in the proliferative neuroepithelium [25,26]. Caspase 9 is activated within a complex known as the apoptosome, where the pro-caspase 9 interacts with Cytochrome c and Apaf-1 [27]. *Cytochrome c *deficient mice die at E8.5 [28], however the majority of *Apaf-1 *mutant mice survive up to E18.5. These mice exhibit defects in the brain, inner ear and digits [29-31]. The middle ear can therefore be analysed in *Apaf-1*, *Caspase 3*, and *Caspase 9 *mutant mice.

To knock down activation of all caspases, an *in vitro *approach must be applied and pharmacological inhibitors binding to the active site of activated caspases are generally used [32]. We have therefore devised a method for culturing the middle ear in the presence or absence of caspase activity. By these two methods the role of apoptosis in formation of the incudomalleal joint can be analysed.

In the digits, developing cartilage recruits neighbouring cells into the cartilage creating high cell density in these regions [33]. It was therefore possible that the *Sox9*/*Type II collagen *expressing cells in the presumptive joint might migrate out of the forming joint into the body of the malleus or incus. To test this we labelled cells in the forming incudomalleal joint region and developing malleus with the lipophilic dyes DiO and DiI, and observed the movements of labelled cells as the joint formed in culture.

Alternatively, the *Sox9*/*Type II collagen *positive cells might remain in position and downregulate expression of these genes. To investigate this possibility we have utilised *Type II collagen *lacZ reporter mice, where the stable lacZ protein marks cells that express *Type II collagen *and also those that have recently switched off *Type II collagen*.

By these methods we hope to determine what processes are involved in early joint development in the middle ear.

## Methods

### Animals

Wild type and *Type II collagen *LacZ reporter mouse embryos were dissected out at embryonic day (E) 13.5 to 18.5.

*Caspase 3*/C57BI/6J mutant mice were provided by Dr. S. Lakhani (Howard Hughes Medical Institute, Yale University School of Medicine, New Haven, USA).

*Caspase 9*/C57BI/6J mutant mice were provide by Prof. K.A. Roth (Department of Neuropathology, University of Alabama, USA).

*Apaf-1 *mutant mice were generated as described in [31].

All research on animals follows internationally recognized guidelines. Use of genetically modified organisms was approved by the local Genetic Modification Safety Committee (GMSC).

### Middle ear explant culture system

Middle ear regions were dissected from the lateral parts of mouse embryonic heads, and placed on Millipore filters supported on metal grids over medium using a modified Trowell method [34]. Culture medium consisted of DMEM (Sigma) supplemented with glutamine, pen/strep and 10% fetal calf serum. Fresh medium was delivered every 24 h and cultures were kept at 37°C, 5% CO_2 _for up to 4 days.

### Caspase inhibition

The pharmaceutical inhibitor of general caspase Z-VAD-FMK (R&D Systems) was dissolved in ACS DMSO (Sigma Aldrich) and added to the fresh culture medium at a final concentration of 200 μM. Control medium was supplemented by 1%DMSO corresponding to the amount of DMSO in the inhibitor solution, as has been used in previous publications [35]. Culture medium was changed every day for 3 days.

Biotinylated pan caspase inhibitor (R&D Systems) was exploited to check the distribution of the inhibitor throughout the explant. The pan caspase inhibitor was applied in the same way as the general one and its distribution was traced in histological sections after addition of streptavidin-AP (BD Biosciences) and NBT/BCIP colour reaction, showing successful spread of the inhibitor (data not shown).

The effect of the inhibitor on apoptosis was tested in developing limbs where the interdigital tissue is known to disappear by caspase dependent apoptosis [36]. Front limbs from E12.5 mouse embryos were cultured simultaneously and in the same way as the middle ear cultures for 24–72 hours. Formation of digits and interdigital space was documented microscopically.

### Staining

Embryos were fixed in 4% paraformaldehyde and dehydrated to 100% ethanol. Paraffin wax sections were cut at 8 μm and split over 5 to 6 slides. Serial sections were stained with hematoxylin/eosin to visualize the early tissue morphology or with Sirrus red and Alcian blue to view the developing bones and cartilage.

*Type II collagen *LacZ reporter mice were fixed and stained with β galactosidase in wholemount as described in [37].

### Proliferation assays

#### PCNA (proliferating cell nuclear antigen)

A polyclonal PCNA antibody (Santa Cruz) was applied for 1 hour (room temperature) after pretreatment in 0.01 M citric buffer (microwave irradiation for 10 mins). Application of the secondary antibody (labeled with horse radish peroxidase – HRP) for 30 mins was followed by a colour reaction using 3',3 '-diaminobensidine (DAB) as the substrate.

#### Anti-phosphohistone H3 immunohistochemistry

Embryos were fixed in 5% acetic acid in ethanol overnight at 4°C, dehydrated through a graded series of ethanol, embedded in paraffin wax and sectioned. Sections were processed for antibody staining using rabbit anti-phosphorylated histone 3 (1:200 dilution, Upstate Biotechnology, Lake Placid, NY) as described previously [38]. The antibody targets were visualized with Alexa-fluor 568-goat-anti-rabbit-IgG (1:500 dilution, Molecular probes, Leiden, the Netherlands). Slides were mounted in Slowfade Gold antifade reagent with DAPI (Molecular Probes) according to manufacturers instructions and visualized by fluorescence microscopy (Carl Zeiss, New York).

### Apoptosis evaluation

#### TUNEL assay

An ApopTag Peroxidase In Situ Apoptosis Detection Kit was used to detect apoptotic DNA breaks in individual cells (Chemicon). For the Colour reaction a DAB Substrate Kit was used, with Nickel staining to give a black colour (Vector Labs). Eosin was used for counterstaining of the sections.

#### Morphological criteria

Nuclear condensations and formation of apoptotic bodies were evaluated additionally to the TUNEL test [39].

#### Cell Labelling

DiI (1,1' dioctadecyl-3, 3,3',3'-tetramethyl-indocarbocyanine perchlorate) and DiO (3,3'-dioctadecyloxacarbocyanine perchlorate) were used as cell lineage labels. A stock solution of DiI (Cell tracker- Molecular probes) (0.5%) was made in 100% ethanol and diluted 1:9 with 0.3 M sucrose on the day of use. For the DiO labelling, a ready-made Vybrant DiO cell-labelling solution was used (Invitrogen). The Dil solution was injected into the developing malleus of dissected lower jaws using a mouth pipette connected to a pulled glass needle. By a similar method DiO was injected into the presumptive joint region between the malleus and incus. The cultures were then moved to explant culture dishes and photographed under light and dark field. Cultures were left to develop for 48 hours and photographed at 24 hour intervals.

### Type II collagen Immunohistochemistry

Type II collagen immunohistochemistry was performed using 11–116B3 antibody (Developmental Studies Hybridoma Bank) on paraffin wax sections. To enhance the signal, slides were microwaved in 0.01 M citrate buffer [40], and treated with Chondroitinase ABC at 0.25 U per ml and Hyaluronidase at 1.45 U per ml at 37°C for 45 minutes (Sigma). The collagen antibody was used at a dilution of 1:100.

### Radioactive *in situ *hybridisation

Radioactive *in situs *were performed using 35S as described [41]. *Gdf5 *probe was linearised with HindIII and transcribed with T7.

## Results

### Proliferation in the incudomalleal joint

**Figure 1:**
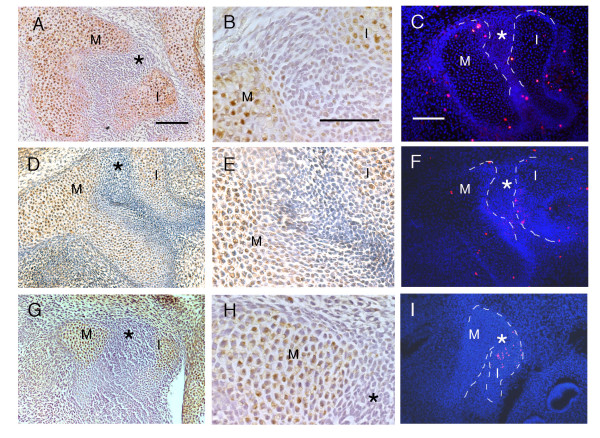
**Proliferating cells are not observed in the developing joint region**. (A, B, D, E, G, H) PCNA staining of sagittal sections through the middle ear. Proliferating cells stained brown against a blue background. (C, F, I) Anti-phosphohistone H3 immunohistochemistry of sagittal sections through the middle ear. Cells outlines by DAPI (blue). Proliferating cells label as bright red spots. Ossicles are outlined with white dashes. (A-C) E15.5. (D-F) E14.5. (G-I) E13.5. (A, D) Malleus and incus. Joint region marked by *. (B, E) High power view of the joint region, showing no positively stained cells compared to the adjacent malleus and incus. (C, F) H3 positive cells are located within and around the ossicles but not in the joint region (*). (G) United malleus and incus. Presumptive joint region marked by *. (H) High power view of the malleus and presumptive joint region, showing no positively stained cells in the presumptive joint (*) compared to the adjacent malleus. (I) H3 positive cells are located within the incus but not in the presumptive joint region (*). M = malleus and I = incus. Scale bars in (A, B, C) = 100 μm.

Very few PCNA positive cells have been observed in the immediate layer of developing joints in the limb [20,42-44]. The joint region is thus thought of as a region of low proliferation. To see whether this situation is mimicked in the middle ear we looked at the distribution of proliferating cells during early formation of the incudomalleal joint. At E14.5 and E15.5 the malleus and incus are seen as separate structures with a clear joint in between. Using PCNA, the level of proliferation was high in the head of the malleus and incus but the joint region was negative (Fig. 1A,B,D,E). PCNA has a long half-life leading to its continuous expression in cells that are not actively dividing [45]. We, therefore, supplemented our results by using anti-phosphohistone H3 immunohistochemistry. The H3 antibody detects phosphorylation of histone H3, which is high during chromosome condensation as the cell enters the M-phase and has a short half-life. As expected, only a few cells were observed as positive in the middle ear using this method compared to using PCNA. The positive cells were concentrated in and around the ossicles but again excluded from the developing joint (Fig. 1C,F). Labelled cells were also located around the circumference of the eye as previously published using this method, indicating that the assay was working [38].

At E13.5 the malleus and incus are still continuous [1]. At this early stage using PCNA, proliferation was observed in the malleus and incus but the forming joint region was negative, reflecting the situation observed at later stages (Fig. 1G,H). Using the H3 antibody at this stage, the incus showed positive proliferating cells but the presumptive joint region appeared negative (Fig. 1I). The malleus also appeared negative, using the antibody to H3, perhaps indicating a time delay between proliferation of the two ossicles.

### Apoptosis in the incudomalleal joint

**Figure 2:**
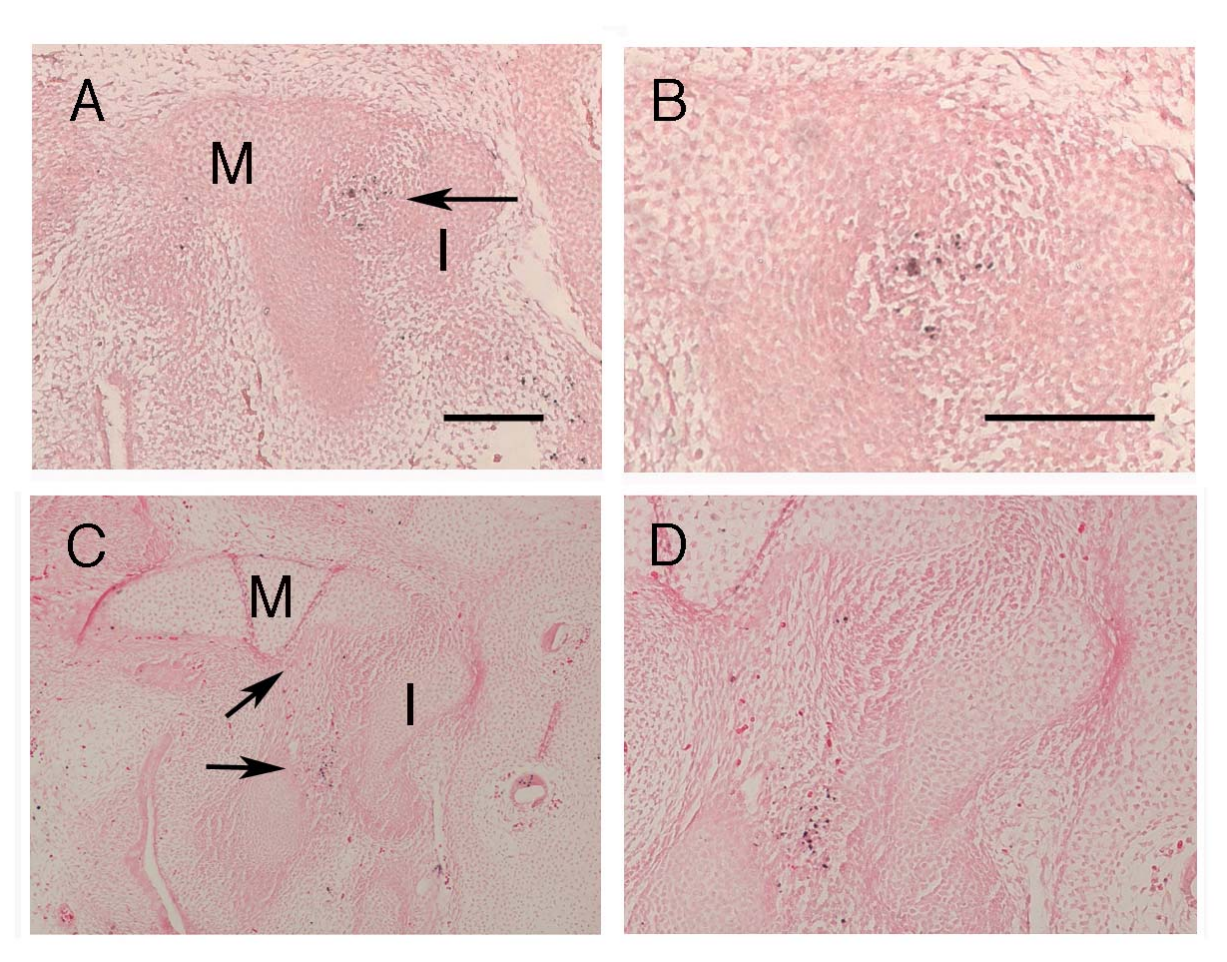
**Apoptotic cells are located between the malleus and incus**. TUNEL staining of middle ear sections in sagittal plane at E13.75 (A, B) and E15.5 (C-D). (B, D) High power views of (A, C). (A, B) Positive cells (black) are located under the head of the malleus as the joint starts to form. (C-D) Positive cells are located between the malleus and incus. Arrows point to TUNEL positive cells. Scale bars in (A, B) 150 μm. M = Malleus. I = Incus.

A TUNEL assay was performed to detect nuclear DNA fragmentation in the developing joint from E13.5 to E15.5. Additionally, morphological criteria of apoptotic cells (nuclear condensation, apoptotic bodies) were evaluated. At E13.75, as the malleus and incus are starting to separate, a clear region of apoptotic cells was visible in a whirl underneath the head of the malleus (N = 4) (Fig. 2A,B). At E14.5 and E15.5, when the joint is clear histologically, a few positive cells were located in this same region under the head of the developing malleus, with a line of positive cells running between the malleus and the incus (Fig. 2C,D and data not shown) (N = 6). TUNEL positive cells were also observed in the external auditory meatus and in the inner ear, where apoptosis has been previously described, confirming that the method was successful [30,46] (data not shown).

To investigate the functional significance of these areas of apoptosis for middle ear development, we investigated middle ear development in mice where different components of the apoptotic machinery had been knocked out, and used an explant culture system to block the action of all caspases in this region.

### Pro-apoptotic molecules *Apaf-1, Caspase 3 *and *9 *are not involved in the initiation of the incudomalleal joint

**Figure 3:**
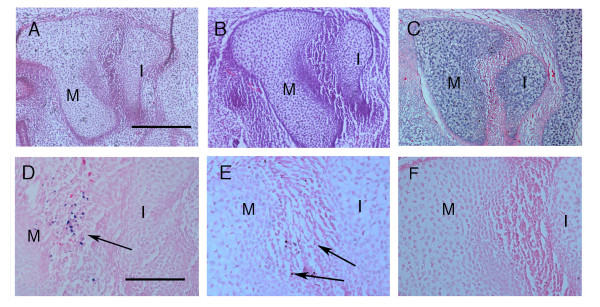
**Loss of *Caspases 3, 9 *or *Apaf-1 *has no effect on the formation of the incudomalleal joint at E15.5**. Sagittal sections of middle ears stained with hematoxylin and eosin at E15.5. (A) The middle ear ossicles in the wildtype. (B) The middle ear ossicles in the *Apaf-1 *mutant. No defect in the initiation of the joint is observed. (C) The middle ear ossicles in the *Caspase 3 *mutant. No defect in the initiation of the joint is observed. (D) TUNEL staining between the middle ear ossicles in a wildtype. (E) TUNEL staining of the middle ear ossicles in an *Apaf-1 *heterozygote. Positive cells are found in between the two ossicles as in the wildtype. (F) TUNEL staining of the middle ear ossicles in an *Apaf-1 *homozygote mutant. The area is negative for TUNEL positive cells. M = malleus and I = incus. Scale bar in (A) = 200 μm. Scale bar in (D) = 100 μm.

**Figure 4:**
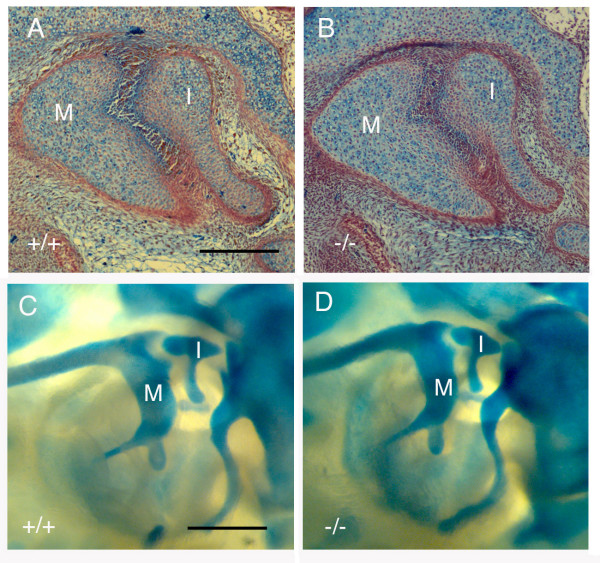
**Joint formation appears normal at E17.5 in *Apaf-1 *mutants**. (A) Trichrome stained section of incudomalleal joint at E17.5 in an *Apaf-1 *wildtype littermate. (B) Trichrome stained section of incudomalleal joint at E17.5 in an *Apaf-1 *homozygote mutant. (C) Skeletal preparation with alcian blue staining of *Apaf-1 *wildtype littermate. (D) Skeletal preparation with alcian blue staining of *Apaf-1 *homozygous mutant. No defect in shaping of the articulation is observed. M = malleus. I = Incus. Scale bar in (A) = 200 μm. Scale bar in (C) = 400 μm.

Mutant mice are a great tool to study the consequences of a particular apoptotic deficiency *in vivo*. *Apaf-1, Caspase 3 *and *Caspase 9 *mutants were analysed at E15.5 to reveal any consequences of their deficiency on early development of the middle ear joint. These embryos displayed a variety of craniofacial defects, with a number showing obvious overgrowth of the brain, as would be expected from previously published observations [31]. By E15.5 the joint region between the malleus and incus is clearly visible in section in wildtype mice (Fig. 3A). The malleus and incus were found completely separated, and no cartilage-like cells were evident in the joint in either the wildtype or mutant embryos (Fig. 3A–C and data not shown) (*Caspase 3 *and *9 *mutants N = 3; *Apaf-1 *mutants N = 4). In order to confirm the absence of apoptosis in these mutants a TUNEL stain was carried out in the *Apaf-1 *mutants. No TUNEL positive cells were observed in the middle ear region, compared to a heterozygous littermate or wildtype, thus confirming that apoptosis has been inhibited in these mutants (Fig. 3D–F). To confirm the lack of phenotype in the mutants, sections were carried out on *Apaf-1 *mutants at E17.5 and E18.5, once the shape of the articulation is well formed (N = 6). In section the incudomalleal joint appeared normal, but the shape of the articulation showed subtle variations in some specimens while in others it appeared identical to that of a wildtype littermate (Fig. 4A,B). This difference might relate to slight variations in the angle of section, rather than indicate a true defect, and so to confirm the result skeletal preparations of mutant heads were examined. In these no obvious defect was observed in the shape of the articulation and the body of the malleus and incus appeared normal (Fig. 4C,D) (N = 4). Loss of apoptosis, therefore, did not appear to disrupt joint formation or gross joint morphology. To confirm this an *in vitro *explant culture approach was introduced to inhibit all caspases in cultures of the middle ear.

### Caspase inhibition in middle ear explant culture

**Figure 5:**
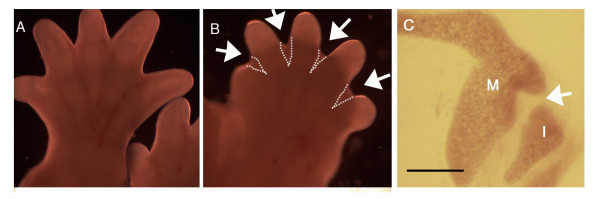
**Inhibition of caspases has no effect on the formation of the incudomalleal joint**. (A) Limb grown for 24 hours in 1% DMSO control medium. (B) Limb grown for 24 hours with Z-VAD-FMK caspase inhibitor. Arrows in (B) point to interdigit webbing in the limbs treated with inhibitor. (C) Middle ear culture grown for three days with Z-VAD-FMK. Arrow points to the incudomalleal joint. M = malleus and I = incus. Scale bar (C) = 200 μm.

Pharmaceutical inhibitors bind to the active site of activated caspases and are routinely used to inhibit caspase activity in vitro [35,36]. For our explant culture we used a modified method of *ex vivo *middle ear culture [47]. This technique allows a clear observation of the middle ear structures as they develop over several days. In order to demonstrate the effectiveness of apoptosis inhibition using the general caspase inhibitor, developing front limbs at E12.5 and E13.5 were cultured *ex vivo *for 24 hours. Interdigital tissue regression was prevented in the caspase inhibitor samples compared to the controls (Fig. 5A,B) (N = 12).

Middle ears were dissected out at E12.5, several days prior to any morphological sign of a joint. These were then cultured for 3 days until a clear joint could be observed between the malleus and incus in control cultures. In cultures treated with the general caspase inhibitor a normal joint was observed, free from cartilaginous cells (Fig. 5C) (N = 4).

### Cell migration

**Figure 6:**
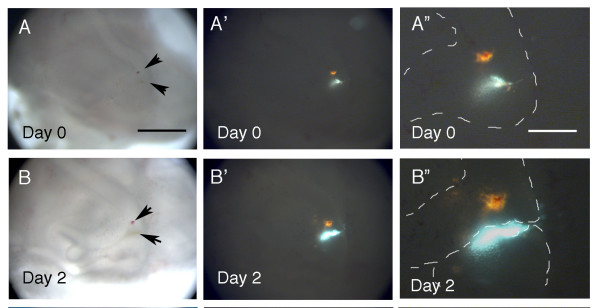
**Cells do not migrate into the malleus from the developing incudomalleal joint**. Middle ear cultures labelled with DiI in the malleus (seen as orange) and DiO in the presumptive joint region (seen as blue). (A, B) Bright field. (A', A", B', B") Dark field image. (A-A") Culture 1 hour after labelling, showing distinct DiI and DiO dots. (A, A') Light and dark field images, same magnification. Arrows in (A) point to the DiI and DiO spots. (B-B") Same culture 2 days after labelling. The malleus and incus are now distinct, with the DiO label located in the joint region. No spread of the DiO labelled joint cells towards the DiI labelled malleus is observed. The DiO label has extended caudally, however, as the joint extends in this direction. (B, B') Light and dark field images, same magnification. Arrows in (B) point to the DiI and DiO spots. Malleus and incus are outlined by white dashes in (A" & B"). Scale bar in (A) = 300 μm. Scale bar in (A") = 100 μm.

Utilising the same middle ear explant culture system, we investigated whether cells in the presumptive joint region migrated away from the developing interzone and were recruited into the ossicles. Cultures were set up at E13.5, before joint initiation. The ossicles and associated middle ear structures developed well in culture along with neighbouring structures such as the inner ear (data not shown). Double labelling was used to identify any movement between cells in the presumptive joint region and the neighbouring malleus. A single spot of DiO was mouth pipetted in the region of the presumptive joint and a second spot of DiI was placed alongside in the developing malleus (Fig. 6A-A") (N = 10). The movement of these two groups of labelled cells was followed for 48 hours. Over this time period no movement of the DiO labelled cells towards the DiI labelled cells was observed (Fig. 6B-B"). As the presumptive joint region is not clearly defined morphologically, in some cases, a few cells in the presumptive incus were also labelled at the same time as the joint. In these cases labelled cells could be seen incorporated into the developing incus (see Fig. 6B"). Although cells did not appear to migrate from the presumptive joint into the malleus, the joint region itself did change shape and elongated caudally as the ossicles developed. This does not necessarily represent active migration but a re-shaping of the joint region as it is squeezed by the proliferating ossicles on either side.

### Downregulation of cartilage markers

**Figure 7:**
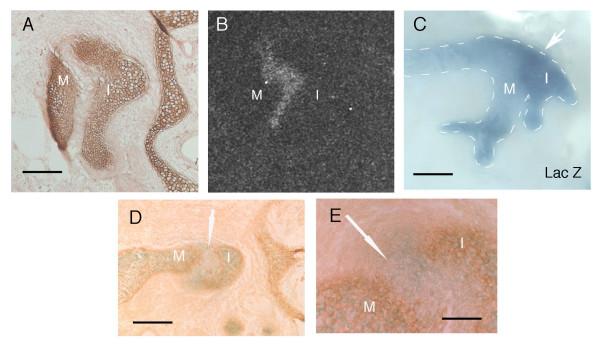
**Downregulation of cartilage fate in the incudomalleal region**. (A) Collagen type II immunohistochemistry of the middle ear ossicles at E14.5. The ossicles appear positive (brown) while the presumptive joint is negative by this stage. (B) *In situ *hybridisation showing the expression of *Gdf5 *in the joint region of the middle ear at E14.5. (C) Dissected middle ear ossicles after wholemount staining for LacZ at E14.5. Strong expression of LacZ is visible in between the ossicles, which appear united (arrow). (D, E) Type II collagen lacZ reporter mouse. Collagen type II immunohistochemistry of the middle ear at E14.5 (brown) and lacZ staining (blue). β gal positive cells are present in the joint region and ossicles, while the Type II collagen protein is only present in the ossicles. Arrows point to joint region. Scale bar in (A) = 100 μm. Scale bars in (C) = 175 μm. Scale bar in (D) = 200 μm. Scale bar in (E) = 70 μm. M = malleus. I = incus.

From the lack of apoptosis or migration out of the forming joint region it would appear that cells in the joint are turning off a cartilage pathway and turning on a joint specific pathway. To test this we used *Type II collagen *reporter mice (where LacZ is inserted into one copy of the *Type II collagen *gene) to take advantage of the fact that the LacZ protein is very stable. At E14.5 *Type II collagen *expression, as assessed by *in situ *hybridisation or immunohistochemistry, is not observed in the joint region (Fig. 7A). Instead joint markers, such as *Gdf5*, are expressed at high levels throughout the joint (Fig. 7B) [1,10]. In contrast, blue cells are still found at high levels in between the two ossicles when the *Type II collagen *lacZ reporter mouse is stained for LacZ at this stage (Fig. 7C). By combining a LacZ stain (blue) and Type II collagen immunohistochemistry (brown), cells that still express Type II collagen will be visualised as both brown and blue, while those cells that have recently turned off Type II collagen will be blue only. We can thus view the expression history of cells in the joint region.

Cells in the joint region at E14.5 expressed LacZ (blue), while the surrounding ossicles expressed both LacZ (blue) and the Type II collagen protein (brown) (Fig. 7D,E). Thus the Type II collagen expressing cells have remained in the same position and downregulated expression of chondrogenic genes.

## Discussion

Apoptosis has been described as a sculptor of development in many mammalian structures and is observed in developing limb joints [16-20]. Thus it seems a good candidate process for removal of *Sox9/Type II collagen *expressing cells to create a joint, and thereby separate the malleus and incus. Apoptotic cells were observed early on between the malleus and incus, however, *Apaf-1*, *Caspase 3 *and *Caspase 9 *mutant mice exhibited normal division of the malleus and incus at E15.5. No middle ear defect was also observed in *Apaf-1 *mutants at E17.5. This is despite the loss of TUNEL positive cells in the mutant. This result was confirmed in explant culture by using caspase inhibitors. Thus formation of the middle ear joint appears independent of apoptosis. It may be, however, that loss of apoptosis is compensated for by cell death through other processes, such as necrosis or caspase independent programmed cell death, such as paraptosis [48]. Paraptosis, like apoptosis, is programmed cell death but is independent of the normal apoptotic machinery [49]. In the limbs of *Apaf-1 *mutants interdigital cell death is initially inhibited but after a delay of a few days, cell death in these regions is observed, resulting in separation of the digits. This interdigital cell death occurs despite no evidence of TUNEL positive cells or overall cell condensation [50]. Thus in the absence of apoptosis, as indicated by TUNEL staining, cells may still be able to die.

Migration is another candidate process that could explain the disappearance of cartilaginous presumptive joint cells. DiI and DiO double labelling was used to trace the movement of cells in the presumptive joint region over 2 days. Results show that although the joint region changes shape, the labelled cells in the joint region did not move towards the DiI labelled cells and were not incorporated into the malleus. This is perhaps unsurprising as chondrogenic precursors have been shown to have a low migration capacity when compared to many other cells types, such as myogenic precursors [51].

By comparing the expression of Type II collagen protein and a LacZ reporter driven by the same promoter, we have shown that the initially chondrogenic cells remain in the incudomalleal joint region and downregulate expression of chondrogenic markers. In this way Type II collagen is turned off and joint markers, such as *Gdf5*, are turned on in the same cells. The signal to trigger this event is as yet unknown. It is thus unclear how cells only in the presumptive joint region turn off *Type II collagen*, while those on either side carry on with a cartilage differentiation pathway. The homeobox gene *Emx2 *may play a role in demarcating the future joint as it is expressed in the incus and presumptive joint but excluded from the malleus, prior to the separation of these two ossicles [1]. In keeping with this role, mutations in *Emx2 *lead to loss of both the incus and joint [52]. Joint markers, such as *Bapx1 *and *Gdf5*, have been shown to turn on before loss of *Sox9 *and *Type II collagen *[1]. It is possible, therefore, that these joint markers act by directly repressing cartilage development. Overexpression of *Gdf5*, however, is unable to induce formation of an ectopic joint, thus although this gene has an important role in joint formation it is not sufficient to repress cartilage development [53,54]. In contrast, overexpression of Wnt signalling is sufficient to induce an ectopic joint [55]. The role of Wnt signalling in formation of the incudomalleal joint and its relationship to the loss of cartilage markers is thus an interesting avenue to pursue. What leads to the induction of joint markers at the future incudo-malleal joint is also unclear. In the chick and zebrafish, *Bapx1 *acts upstream of *Gdf5 *in development of the primary jaw joint, with the expression of *Bapx1 *being controlled by a combination of inhibitory and inducing signals from Fgfs, Bmps and Endothelin [8,9]. Whether a similar combination of signals leads to the restriction of joint markers in the mammalian middle ear is therefore another important area for future study. Such comparative work will allow for further understanding of how cranial joints are initiated and positioned and will focus in on the genetic pathways that may have played a role in changing the pattern of joints during evolution.

## Conclusion

The incudomalleal joint in the mammalian middle ear separates the initially united incus and malleus. The forming joint is characterised by a lack of proliferation, compared to the ossicles on either side, and contains a number of cells undergoing cell death (apoptosis). In the absence of apoptosis, however, no defect in joint formation is observed. Cells in the joint region do not move away from this position but instead downregulate cartilage markers and take on a joint fate, expressing genes such as *Gdf5*. Fusions of the middle ear, as seen in patients with BOR Syndrome or Treacher Collins Syndrome, may therefore occur if presumptive joint cells fail to downregulate expression of chondrogenic genes. Many of the genes underlying such syndromes have been discovered, and so the next challenge is to investigate such possibilities using mouse models.

## Competing interests

The author(s) declare that they have no competing interests.

## Authors' contributions

SA and AST wrote the manuscript. SA performed the majority of the experiments described with the help and assistance of AT, in whose lab the research was carried out. EM performed the PCNA results, provided the Caspase mutant sections and wrote the text relating to apoptosis. CS sectioned *Apaf-1 *mutants and performed the anti-phosphohistone H3 immunohistochemistry. HY provided a range of *Apaf-1 *mutant embryos. All authors have read and approved the final manuscript.
